# Job description and perception of clinical research personnel working in a network of French intensive care units

**DOI:** 10.1186/s13054-024-04900-8

**Published:** 2024-04-11

**Authors:** Mireille Adda, Claire Dupuis, Gérald Gouby, Claude Dubray, Jean Reignier, Bertrand Souweine, Christian Dualé

**Affiliations:** 1 CHU Clermont-Ferrand, Médecine Intensive et Réanimation, Clermont-Ferrand, France; 2 Direction de la Recherche Clinique et des Innovations, CHU Clermont-Ferrand, Clermont-Ferrand, France; 3 Université Clermont-Auvergne, Clermont-Ferrand, France; 4 CHU Nantes, Médecine Intensive et Réanimation, Nantes, France; 5 CHU Clermont-Ferrand, Centre d’Investigation Clinique (INSERM CIC1405), 58 Rue Montalembert, 63000 Clermont-Ferrand, France

**Keywords:** Research personnel, Intensive care, Job stress, Job satisfaction

## Abstract

**Background:**

There is a lack of information about the organisation and management of clinical research personnel in Europe and of their professional activity in intensive care. We therefore conducted a cross-sectional survey among personnel currently working in a French intensive care research network that involves 41 centres nationwide. The aim of the survey was to describe the personnel’s personal and institutional organisation and management, their job perception in terms of satisfaction and stress, and suggestions for improvement.

**Methods:**

Over 3 months in 2023, the research personnel received an electronic questionnaire on their personal and professional profile, past and present training, workplace and functions currently performed, personal knowledge about job skills required, job satisfaction and stress by as measured on a rating scale, and suggested ways of improvement.

**Results:**

Ninety seven people replied to the questionnaire (a response rate of 71.3%), of whom 78 (57.3%) were sufficiently involved in intensive care to provide complete answers. This core sample had profiles in line with French recruitment policies and comprised mainly Bachelor/Master graduates, with nurses accounting for only 21.8%. The female to male ratio was 77:23%. Many responders declared to have a shared activity of technician (for investigation) and assistant (for quality control). More than 70% of the responders considered that most of the tasks required of each worker were major. Figures were much lower for project managers, who were few to take part in the survey. On a scale of 10, the median of job satisfaction was 7 for personal work organisation, 6 for training and for institutional organisation, and only 5 for personal career management. The median of job stress was 5 and was inversely correlated with satisfaction with career management. Respect of autonomy, work-sharing activity between investigation and quality control, a better career progression, financial reward for demanding tasks, and participation in unit staff meetings were the main suggestions to improve employee satisfaction.

**Conclusion:**

This nationwide survey provides a new insight into the activity of French clinical research personnel and points to ways to improve the quality and efficiency of this workforce.

**Supplementary Information:**

The online version contains supplementary material available at 10.1186/s13054-024-04900-8.

## Background

Clinical research, which refers to scientific studies performed on human subjects with the aim of furthering biological or medical knowledge, is a major branch of medicine [1]. In parallel to research conducted by companies to develop drugs or medical devices, academic research improves knowledge, covering a broad range of fields from epidemiology and pathophysiology to clinical trials. Public hospitals are an ideal setting for academic research, in view of their objectives, medical expertise, technical resources, and specific funding. In this context, French academic research has considerably developed over the past decades: about 8000 studies were registered between 2008 and 2017, and during that period the cumulative number of active studies rose from 1493 to 2414 during that period [2, 3].

The scientific and technical aspects of drug development have been addressed by the International Council for Harmonisation (ICH) since 1990 [4], and the ethical and scientific quality standard of clinical trials is internationally covered by Good Clinical Practice (GCP), which is set out in laws and regulations worldwide. To comply with these requirements, French public hospitals—especially those affiliated to universities—have provided significant funding. A survey conducted in 2008 by the French Ministry of Health on jobs under pressure in public hospitals established the profiles of those dedicated to clinical research, highlighted the youth of the workers, and identified factors affecting their development [5]. In 2023, the Ministry of Health updated its definition of clinical research jobs, establishing a list of the tasks required and background training [6]. The three most important jobs were clinical research assistant (CRA), clinical research technician (CRT), and clinical research project manager (CRPM). A description of the jobs and related tasks is given in Table 1.

**Table 1:** Job description of the clinical research personnel as defined by the French Ministry of Health (2023)

Jobs and related tasks	Abbreviation
Clinical research assistant	CRA
Designing and building tools or methods	Tools&Meth
Checking application of rules, procedures, norms, and standards	RPNS
Checking conformity/validity of documents	CVDoc
Checking logistical feasibility of the study	Feasibility
Checking and monitoring the quality of process(es)	Quality
Organising events such as meetings, visits, and specialised committees	Events
Drafting reports on observations/interventions	Reports
Monitoring adverse events	MonAEs

Clinical research technician	CRT
Checking feasibility of the study’s logistical circuits	Circuits
Establishing/updating, and implementing processes, procedures, protocols, and instructions	PPPI
Managing (browsing, collecting, analysing, prioritising, sharing, filing, tracking) data and information	Manage D&I
Informing/advising the caring staff, patients, families, etc.	Communication
Organising data checking before monitoring visits	Pre-visit
Preparing biological sampling, storage, and shipping	PrepBiol
Assessing and presenting the clinical activity of the unit	ClinActiv
Gathering data or information	Gather D&I
Completing documents and files (activity or traceability sheets, etc.)	CompletDoc
Reprography, anonymising results, and transmitting data to the coordinating centre	HandleDoc
Reporting/tracking adverse events	RepAEs
Dispatching study documents (recording, sorting, processing, distribution, archiving)	DispatchDoc
Pre-analytical treatment of samples	Samples

Clinical research project manager	CRPM
Checking application of rules, procedures, norms, and standards	RPNS
Checking meeting of deadlines (products, files, interventions, etc.)	Deadlines
Supervising team(s), staff management, and implementation	Supervision
Establishing/updating and implementing processes, procedures, protocols, and instructions	PPPI
Drawing up specifications according to customer’s requirements	Customer
Planning activities and resources; control and reporting	Planning
Finding/managing the financial, human, and logistical resources	Resources
Managing grant application files and calls for tender	Grants

Description of the most recently edited definition of the tasks for each main job of the clinical research personnel by the French Ministry of Health (in year 2023); web source: https://sante.gouv.fr/metiers-et-concours/les-metiers-de-la-sante/le-repertoire-des-metiers-de-la-sante-et-de-l-autonomie-fonction-publique/recherche-clinique

**Fig. 1:**
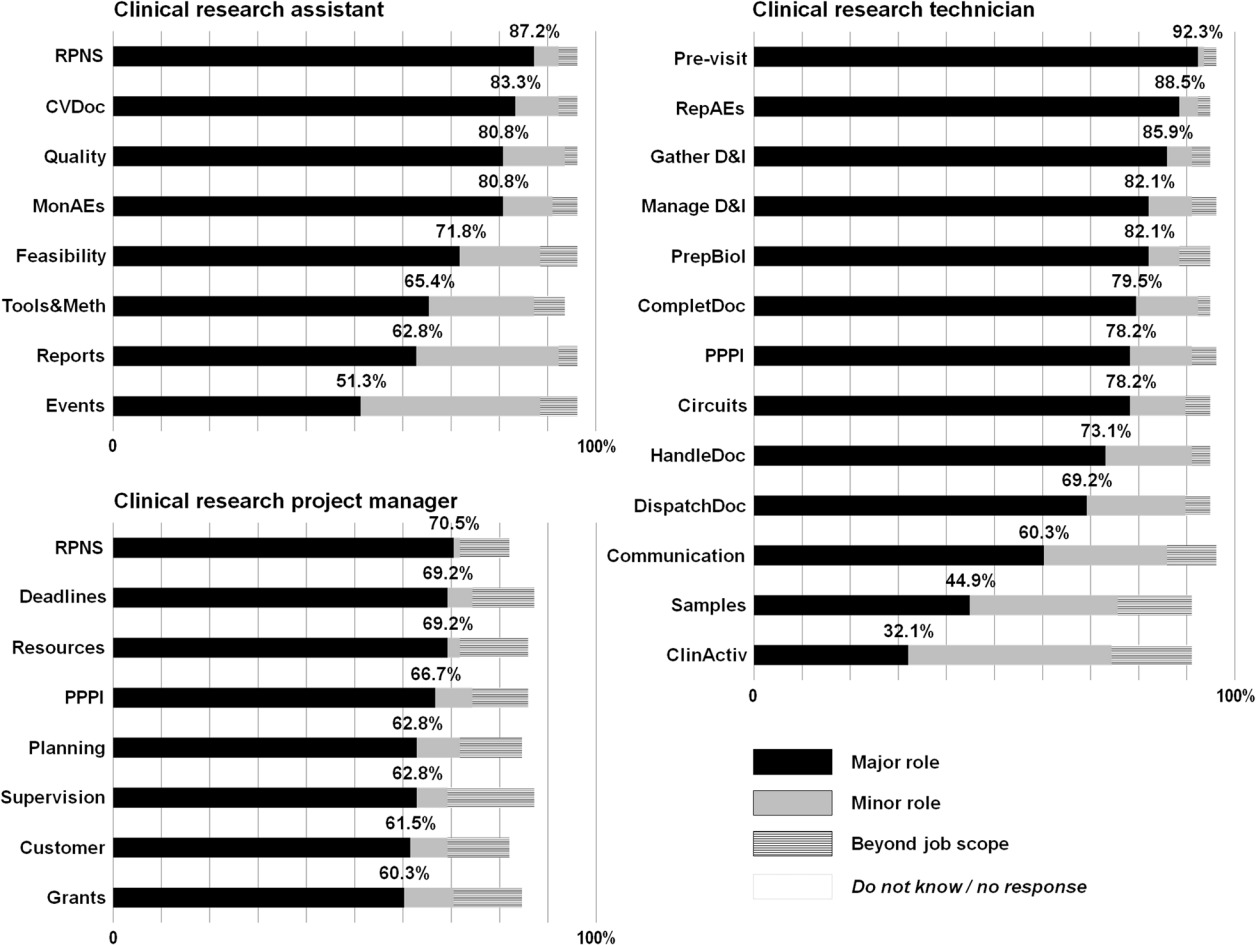
Responses about the personal definition of the respective jobs of CRA (i.e. working on behalf of the sponsor), CRT (i.e. working on behalf of the centre with the investigating team), and CRPM. Each surveyed person had to define each particular task in terms of relationship with the job as either ‘major’, ‘minor’, or ‘beyond job scope’. An optional ‘do not know’ was also offered. For each job, the tasks are ranked by decreasing rate of ‘major’ responses, or rate of [‘major’ + ‘minor’] in case of equally ranked tasks. The response rate per item and per task is shown by a horizontal stacked bar (black for ‘major’, grey for ‘minor’, striped for ‘beyond job scope’, and transparent otherwise). The percentage values appearing over each bar are those which were considered for the ranking mentioned above. The abbreviations used to describe each task are explained in Table 1

NB: the French wording, respectively, for CRA, CRT, and CRPM, is: assistant de recherche clinique, technicien de recherche clinique, and chef(fe) de projet de recherche clinique. The abbreviations (right column) have been created to make it easier to read Fig. 1

However, very little work has been done on the current organisation and management of research personnel in French public hospitals. We therefore conducted a nationwide survey among a representative sample of academic clinical research personnel working in intensive care medicine (ICM). In clinical research, ICM is a particularly productive field, in which France is ranked fourth in the world according to the H-index, either cumulative 1996–2022 or for the sole year 2022 [7]. French ICM research is backed up by several registries held by networks such as OutcomeRea (nosocomial infections), REVA (artificial ventilation), and CRICS-TRIGGERSEP (sepsis). Notably, it has significantly contributed to improving knowledge and treatment of COVID-19 [8, 9]. Over the whole French territory in 2019, there were 5080 beds in about 340 adult intensive care units (ICU), of which 89% were housed in public or subsidised hospitals [10, 11].

We conducted this questionnaire survey within the NUTRIREA network, which currently performs nutritional multicentre clinical trials involving 41 ICUs nationwide [12]. Our aim was to describe the current organisation and management of human resources for clinical research in the network. We focused our interest on the personnel’s professional profile, their current functions and tasks, the common skills they require, and what continuing education and career opportunities are offered. We also aimed to assess the survey sample’s perceptions about their job in terms of satisfaction and stress. Our long-term aim was to identify ways of improving efficiency and quality and to propose that some of the conclusions drawn could be extended to other medical specialties and other countries.

## Materials and methods

The study was approved by the local ethics committee and complied with the French policy of individual data protection. The cross-sectional closed survey was conducted among the clinical research personnel of the French intensive care research network involved in the NUTRIREA3 study, a French multicentre trial [13]. The 41 ICU centres of the network, of which 27 are affiliated to universities, belong to public hospitals. The coordinating team of this network identified their CRAs, CRTs, and CRPMs and provided the study coordinator (M.A.) with a list of this personnel with their e-mail addresses and phone numbers. The study coordinator then contacted each person by email or telephone to explain the aims and procedure of the survey.

Data were collected by REDCap electronic data capture tools in accordance with the French and European laws and regulations on data protection. Informed consent was implicitly given when the responder agreed to create an account. The data were transferred to a separate datasheet (Microsoft Office Excel 2013, Redmond, WA, USA). If the questionnaire was not completed within 6 weeks, a reminder was sent via email and then up to two other reminders were issued when necessary. The survey started on 18/01/2023 and ended on 03/04/2023.

The survey was developed by the authors following a stepwise procedure. First, the study coordinator interviewed several CRAs, CRTs, staff managers, or clinical researchers in our institution who are currently involved in the job definition, recruitment, and coordination of clinical research personnel. From the content of these interviews, the questionnaire was then drafted by the project leader and the methodologist (C.D.). After correction by the other authors, a new version was drafted and entered in the data capture system and then pre-tested by three CRAs/CRTs currently working in our university hospital, who created an account for this purpose. After corrections of wording or presentation, a final version was validated by the authors.

The questionnaire is fully described in Additional file 1. After a preliminary section presenting demographic and workplace details, the following sections surveyed, in order of presentation: (i) the job history (training and experience); (ii) the perception of the tasks related to each of the three main clinical research jobs in France (i.e. CRA, CRT, and CRPM); (iii) the current organisation of the job in detail; (iv) the personal definition of the three above-mentioned jobs (supported by the job description published on the Ministry of Health website, see Table 1) [6]; (v) the perception of the positive and negative aspects of the job; (vi) how the person’s career was managed and how it is planned; and (vii) various quantitative and qualitative questions about job stress and job satisfaction within several domains such as organisation, training, and career. The responders were freely invited to make suggestions on how to improve the job. In the current report, only information relevant to an international readership will be presented and hence specifically French aspects of organisation, for example, will not be dealt with.

The dataset was anonymous, and responders were given clear instructions not to duplicate entries. Duplicates were identified by checking if the demographics and workplace variables contained exactly the same data; in such cases, the most recent entry was retained. Missing data were not replaced, and if, for one given responder and within one given domain, attrition made impossible a clear description, the block of data could be discarded from analyses, if appropriate. The primary aim of the statistical analysis was descriptive. Numerical data were expressed as mean ± SD or as quartiles, depending on their distribution. Nominal data were expressed as headcount and proportion of total.

Complementary analyses were conducted to study certain interrelations between variables of interest. Firstly, we studied the interrelations within the four indicators of job satisfaction and job stress and as all were assessed by a numerical rating scale from 0 to 10, we performed Spearman’s correlation analyses. Each correlation was expressed using its own *ρ* coefficient, and the difference between *ρ* and 0 was tested. The inferences were made only to highlight the strongest associations, and the type-I error inflation was not corrected. Secondly, we studied the possible influence of several factors on each indicator of job satisfaction and stress using either Spearman’s correlation analyses (when the factor to test was a numerical variable) or a Mann–Whitney test (when the factor to test was a binary variable). To harmonise the effect sizes, binary variables were also treated as numerical, i.e. yes/no variables became 1/0, and “sex” became “male” (1/0). The factors we tested were sex, age group, civil status, type of hospital, educational level, number of years of experience in clinical research, type of current contract, size of the research team in the unit, amount of weekly work, number of studies as supervisor, and level of externality. The last variable was created by estimating the ratio of studies with an external sponsor versus those sponsored by one’s own institution. Thirdly, we studied the relationship between job stress and the responders’ most appreciated and disliked aspects of the job. For this assessment, the responders were categorised according to the aspect they had ranked first (separately for the appreciated positive aspects and the disliked negative ones). We aimed at avoiding small classes, and so for those responders who ranked first an aspect rarely ranked as such by others, the firstly ranked aspect was replaced by the secondly ranked. Finally, job stress was tested against the most appreciated and disliked aspects by a one-way ANOVA followed by a post hoc Tukey’s test.

Statistical analyses were performed with XLStat (Addinsoft, Paris, F). Figures were generated with Microsoft Office Excel 2013 and PowerPoint 2013 (Microsoft, Redmond, USA), and Photoshop Elements 7.0 (Adobe, San Jose, USA).

## Results

The initial mailing list contained 151 addressees, of whom 15 did not send a return receipt. The remaining 136 created an account on the REDCap platform: 97 (71.3%) answered the questionnaire, 78 with full completion (57.3% of the 136), and 19 only the initial section dealing with demography and general characteristics of the job setting. No duplicate entries were identified. The data suggested that many of the partial responders were not heavily involved in ICU, as this was only a very small part of their current activity, hence all the following results were obtained from the main core of 78 full responders, whose general characteristics are given in Table 2. There were no missing data for this sample. Most of the responders were women, middle-aged, Bachelor or Master graduates, had good professional experience in clinical research (about half of which was in ICU), were mostly in full-time employment, and shared the activities of CRT (their main function) and CRA. The number of CRPMs was much lower. Of note, 21.8% of responders were Bachelor graduates in nursing. Full responders also included one dietician, one psychologist, and two laboratory technicians. The most common place of work was a single ICU in a university hospital, but work activities could be spread over several units and not exclusively in ICUs. The type of studies the responders were involved in varied according to setting or sponsoring: the most common activity profile was concomitant work on a few studies sponsored by the employer institution and on various studies with external sponsorship, or of a multicentre nature. The 19 responders who did not fully complete the survey were not greatly different from the core sample, but were slightly older, less often graduates, and more likely to be affiliated to a general hospital (Additional file 2).

**Table 2:** Description of the survey sample (*N* = 78)

Demography	
Female	60 (76.9)
Age group	
< 30	7 (9.0)
30–39	34 (43.6)
40–49	26 (33.3)
50–59	10 (12.8)
> 60	1 (1.3)
In a partnership	59 (75.6)
Number of dependent children (including if joint custody)	
0	26 (33.3)
1	11 (14.1)
2	29 (37.2)
3 or more	12 (15.4)
Educational level	
French baccalauréat (or equivalent)^a^	75 (96.2)
Current highest level of education^b^	
No degree	8 (10.2)
Bachelor degree	26 (33.3) ^c^
Master degree	37 (47.4)
PhD	7 (9.0)
Specific CRA/CRT diploma when taking up the first job in the hospital	37 (47.4)
Experience in clinical research	
Number of years’ experience	
In clinical research, all functions combined	10 [6–13]
In the current workplace, all functions combined	10 [4–12]
In ICU, all functions combined	5 [3–9]
Time spent working in clinical research versus time spent working in the institution	
Lower	26 (33.3)
Equal	36 (46.2)
Greater	16 (20.5)
Time spent working in clinical research versus time spent working in ICU	
Lower	45 (57.7)
Equal	33 (42.3)
Greater	0 (0.0)
Share of past activity in clinical research (% of the whole spent time) ^d^	
As a CRA	37.5%^e^
As a CRT	45.2%^e^
As a CRPM	3.2%^e^
In another function	15.2%^e^
Job description	
Type of hospital	
General	23 (29.5)
University	52 (66.7)
Private but non-profit-making	3 (3.8)
Current contract	
Temporary with definite duration	16 (22.2)
Temporary with indefinite duration	33 (45.8)
Tenure	23 (31.9)
Size of the ICU as workplace (number of beds)^f^	20 [16–25]
Number of CRAs/CRTs currently working in the unit (in FTE)	2 [1–4]^g^
Number of CRPMs currently working in the unit (in FTE)	0.3
Number of ongoing studies in the unit with external sponsorship	12 [6–17]
Number of ongoing studies in the unit, sponsored by own institution	4 [1–6]
Amount of weekly work (% of a FTE)	
< 20%	1 (1.3)
50–60%	2 (2.6)
80–90%	12 (15.4)
100%	63 (80.8)
Number of weekly teleworking days	
0	49 (62.8)
1	20 (25.6)
2–3	6 (7.7)
4–5	3 (3.8)
Share of current activity in clinical research (% of the whole spent time)^d^	
As a CRA	31.4^e^
As a CRT	47.0^e^
As a CRPM^h^	15.4^e^
In another function	6.5^e^
Activity spread over several departments/units	28 (38.9)
If yes, number of departments/units	5 (3–9)
If yes, percentage of time spent in ICU	50 [24–71]
Number of ICUs where current activity is taking place	
1	23 (82.1)
2	2 (7.1)
3	3 (10.7)
Average number of studies supervised at a given time, sponsored by own institution	
0	11 (15.1)
1–2	28 (38.4)
3–4	16 (21.9)
> 4	18 (24.7)
Average number of studies supervised at a given time, with external sponsor	
0	2 (2.7)
1–5	15 (20.5)
5–10	16 (21.9)
> 10	40 (54.8)
Average number of multicentre studies supervised at a given time	
≤ 10	34 (46.6)
11–25	31 (42.5)
> 25	8 (11.0)
Ditto, in quantitative terms	11 [7–18]
Location of activity (% of whole current clinical research activity)	
In the unit^i^	32.8^e^
In office ^j^	61.6^e^
Other	6.0^e^
Attendance at department staff meetings	45 (57.7)^k^
Work at night or on weekends	22 (30.1)
Report of appraisal meetings with a supervisor	61 (84.7)^l^
Prospects for career development	22 (30.6)

General description of the surveyed sample (only the 78 participants who fully completed the survey (see Results section for details). Numerical data are expressed as median (1st quartile–3rd quartile) or mean (in italics) when more appropriate. Nominal data are expressed as headcount (%). Abbreviations: CRA: clinical research assistant; CRPM: clinical research project manager; CRT: clinical research technician (see Table 1 for job definition); FTE: full-time equivalent, i.e. the legal maximum working time, which is currently 35 h a week in France: 1 FTE or 100% FTE corresponds to this duration; ICU: intensive care unit. ^a^Equivalent to ‘A’ level in the UK or High School Diploma in the USA; ^b^in the French university system, the three levels of graduation (college) are *licence* (Bachelor, 3 years), *master* (2 years), and *doctorate* (e.g. PhD); ^c^including 17 Bachelors in Nursing (21.8% of the whole sample); ^d^whatever the amount of weekly work; ^e^mean values are preferred in order to reach a sum of 100%; ^f^the main ICU if activity shared between several ICUs/departments; ^g^max = 30; ^h^or clinical research coordinator; ^i^e.g. management of inclusions, patient follow-up, etc. ; ^j^e.g. data entry, file management, drafting of protocols, articles, procedures, etc. ; ^k^weekly for 35.6% of the whole sample; ^l^annually for 73.6% of the whole sample

Figure 1 shows how the responders defined the jobs of CRA, CRT, and CRPM. As the tasks to be rated within each job were actually related to the job—the aim was not to mislead the responder—responses such as ‘major’ or ‘minor’ relationship can be interpreted as levels of agreement on how the task matches the job definition. Hence, the tasks with the highest agreement with the job criteria of a CRA were “checking application of rules, procedures, norms and standards”, “checking conformity/validity of documents”, “checking and monitoring the quality of process(es)”, and “monitoring adverse events”: the agreement was moderate for “organizing events such as meetings, visits, and specialised committees”. Agreement was high for most of the tasks involved in the job of a CRT, but moderate for “informing/advising the caring staff, patients, families, etc.”, “pre-analytical treatment of samples”, and low for “assessing and presenting the clinical activity of the unit”. Agreement was generally lower for most of the tasks presented involved in the job of CRPM than for the functions of CRAs and CRTs, with 30 to 40% of responses that could be considered as inappropriate (i.e. “beyond job scope”, “do not know” or no response).

**Fig. 2:**
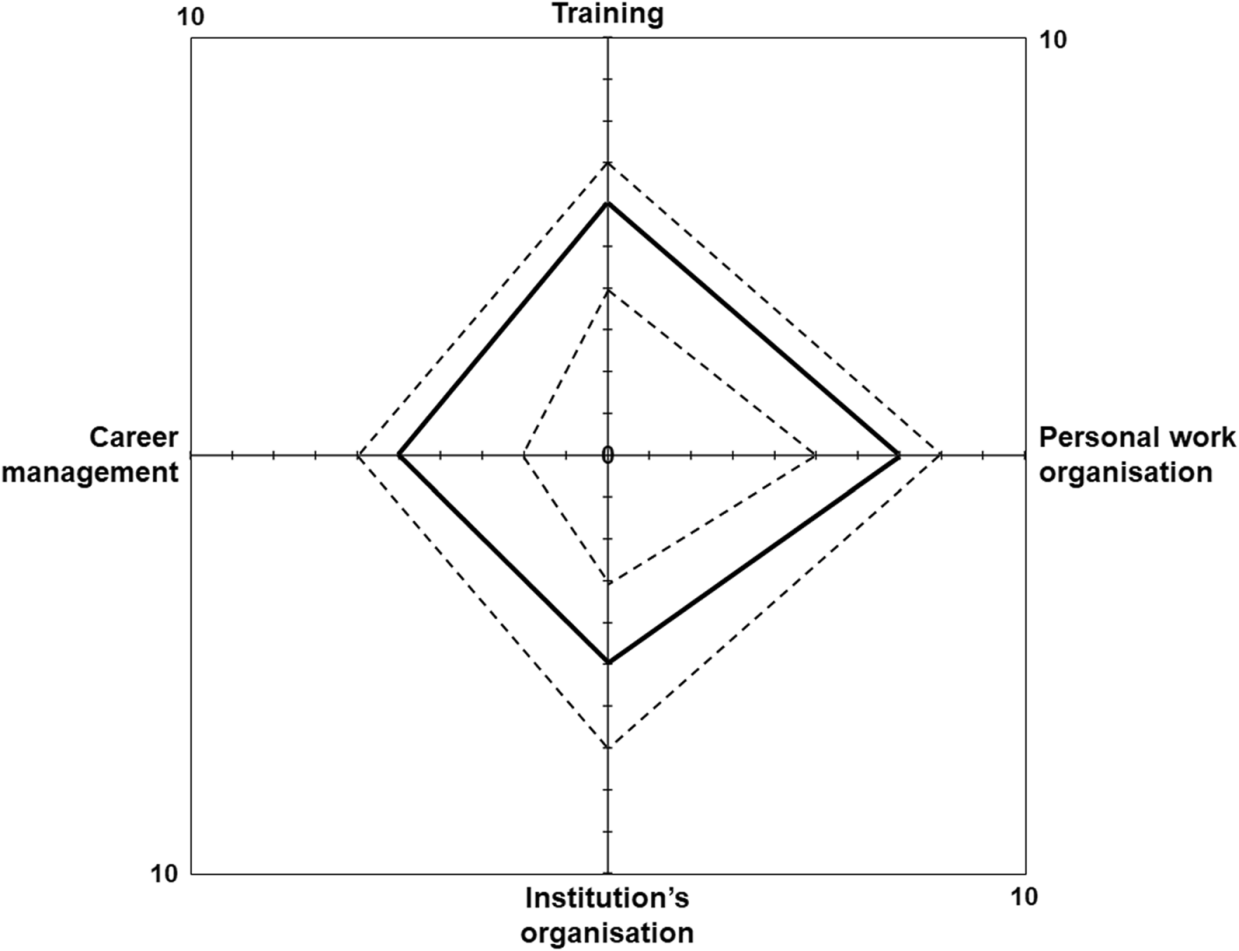
Radar chart of the responders’ job satisfaction (*N* = 78). Satisfaction was assessed with an 11-point numerical scale, from 0 (maximum dissatisfaction) to 10 (maximum satisfaction). Four domains of satisfaction were assessed: training program since joining the institution (‘training’), personal work organisation, institution’s overall organisation of clinical research (‘institution’s organisation’), and personal career management by the institution (‘career management’). The full black lines represent the median values for the sample, and the grey dotted lines represent the 1st (inner) and 3rd quartile (outer)

Table 3 and Fig. 2 show the most important aspects of the responders’ job perception. The most appreciated positive aspect of the job was autonomy, and the most disliked negative aspects of the job were lack of time, and administrative procedures and paperwork. The highest job satisfaction was for personal work organisation (median = 7/10). Satisfaction with training received since joining the institution was slightly lower (median = 6/10), and satisfaction with the institution’s overall organisation of clinical research and personal career management was even lower (median = 5/10). For those responders who were currently taking part in unit staff meetings the perception of their participation was 64% positive, especially because it helped identification or selection of patients for inclusion. For those responders with shared workload (for example partly as a CRA and partly as a CRT), managing multiple tasks was perceived positively, with a high personal satisfaction and professional achievement**.** The median job stress was 5/10, which is average on the scale. Neither the amount of time spent in ICU (vs. other clinical specialties) nor the level of multivalence influenced job satisfaction or job stress (no significant correlation). The responders working in a university hospital were less satisfied with their personal career management than those working in a general hospital (median score = 6 vs. 7, respectively, *p* = 0.029). However, the type of institution did not influence any of the other parameters of job satisfaction and stress.

**Table 3:** Personal job perception (*N* = 78)

About participation in unit staff meetings (valid for 57.7% of the sample)	
Positive perception of the usefulness	47 (64.4)
Reasons for usefulness, ranked from most to least useful	
Facilitating identification or selection of patients for inclusion	1st
Better integration in the team	2nd
Greater knowledge of diseases and care	3rd
Learning/expanding medical vocabulary	4th
Perceived positive aspects of the job (ranked from most to least appreciated)	
Autonomy	1st
Relational aspects	2nd
Working in a team	
Scientific interest	
Organisation and logistical management	5th
Personal development	6th
Perceived negative aspects of the job (ranked from most to least disliked)	
Lack of time	1st
Administrative procedures and paperwork	
Constraints	3rd
Routine	4th
Isolation	
Satisfaction with the training programme since joining the institution^a^	6 (4–7)
About the multivalence of the job (valid for 50.0% of the sample)	
Satisfaction with multivalence	
Dissatisfied	0 (0.0)
Not much satisfied	2 (5.1)
Neutral opinion	3 (7.7)
Satisfied	10 (25.6)
Very satisfied	24 (61.5)
Sense of personal job efficiency^b^	8 (7–8)
About the organisation of clinical research within the institution	
Satisfaction with the institution’s overall organisation^a^	5 (3–7)
Perception of complexity of the institution’s organisation in managing the studies it sponsors^c^	7 (5–8)
Satisfaction with personal work organisation^a^	7 (5–8)
Satisfaction with personal career management by the institution^a^	5 (2–6)
Job stress	
Personal stress in the job^d^	5 (2–7)

^a^Rated on an 11-point numerical scale, from 0 (maximum dissatisfaction) to 10 (maximum satisfaction); ^b^rated on an 11-point numerical scale, from 0 (no efficiency) to 10 (maximum efficiency); ^c^rated on an 11-point numerical scale, from 0 (no complexity) to 10 (maximum complexity); ^d^rated on an 11-point numerical scale, from 0 (no stress) to 10 (maximum stress)

Details of the tasks currently carried out by the responders are given in Additional file 3. The most common method used for screening eligible patients for studies sponsored by an external institution was consultation of the patients’ medical records (62.8%), and the most commonly performed tasks in the office were data entry, responses to queries, and reporting SAEs (84.6%), patient follow-up (70.5%), and drafting procedures (62.8%).

The responders’ suggestions to improve the different aspects of their job are detailed in Additional file 4. Financial reward for demanding tasks was the most often cited (75.6%) as a way to improve career management.

The possible sources of stress identified by the responders are detailed in Additional file 5. The most often cited sources were institutional disregard of the needs of clinical research personnel (50%) and poor recognition of the job within the institution (60.3%).

Table 4 shows how the indicators of job satisfaction and job stress were interrelated. The four domains of job satisfaction were collinear, with mild to moderate positive correlations. There was a high coherence within job satisfaction, with a Cronbach’s α coefficient of 0.795. Job stress was inversely correlated with satisfaction with career management. A similar trend—albeit non-significant—was also observed for the relation between job stress and satisfaction with training and personal work organisation. No relationship was observed between job stress and satisfaction with the institutional organisation of clinical research.

**Table 4:** Spearman correlation matrix of job satisfaction and stress

	Job satisfaction			Job stress
	Personal work organisation	Career management	Institution’s organisation	
*Job satisfaction*				
Training	*0.363*	**0.643**	**0.603**	− 0.207
Personal work organisation		*0.345*	*0.380*	− 0.166
Career management			**0.622**	*− 0.258*
Institution’s organisation				− 0.070

In each case is shown the ρ coefficient for the correlation tested between the variable indicated on the row header and this indicated on the column header. The significant differences with the null value are signalled in Italic; the significant moderate correlations (0.4 to 0.7 or − 0.4 to − 0.7) are Bold. Job satisfaction was assessed with an 11-point numerical scale, from 0 (maximum dissatisfaction) to 10 (maximum satisfaction); job stress was assessed with similar scale, from 0 (minimum stress) to 10 (maximum stress). Four domains of satisfaction were assessed: training programme since joining the institution (‘training’), personal work organisation, institution’s whole organisation for clinical research (‘institution’s organisation’), and personal career management by the institution (‘career management’)

When testing the factors likely to influence job satisfaction, only one relevant relationship was found: satisfaction with training was lower in the university hospitals than in the general hospitals (median score = 6 and 7, respectively, *p* = 0.029). When testing the factors likely to influence job stress, two relevant relationships were found: the size of the research team within the unit negatively correlated with stress (*ρ* = − 0.281; *p* = 0.017), and stress was higher in women than in men (median score = 5 and 2, respectively, *p* = 0.024).

Finally, the responders who cited lack of time as the most disliked aspect had a higher stress score than those who cited administrative procedures and paperwork as the most disliked aspect (median score = 7 and 3, respectively, *p* = 0.008). No relationship was observed between job stress and the responders’ most appreciated aspects of the job.

## Discussion

To the best of our knowledge, this is the first broad description of ICU clinical research personnel in Europe, from a country strongly involved in ICM academic research. Although our observations do not necessarily extend beyond France or to other medical specialties, they identify issues and hypotheses that could be relevant to broader fields.

The most typical profile of the clinical research personnel surveyed was that of a middle-aged woman, a Bachelor/Master graduate working full-time or almost full-time as a CRA or CRT in a university hospital, whose activity was fairly equally shared between the two functions. Training in clinical research had been done before and after hiring and completed by an average professional experience of 10 years. This profile is very similar to that of 98 research coordinators recently surveyed in Australasian ICUs [14]. The high female rate reflects the current sex ratio observed in healthcare professionals [15] and is probably helped by a favourable work-life balance [16]. The age distribution reflects a hiring wave that started about 25 years ago, which along with educational level and job functions is in line with French government policies since the creation in 1992 of clinical research offices in public hospitals, accompanied by increased dedicated funding [2, 5, 17].

To comply with GCP, the French Ministry of Health published in 2021 an index of jobs in the public hospital service, including clinical research [6]. In the index, the main jobs were clearly defined as: (i) launching research protocols and monitoring their quality on behalf of the sponsor (for a CRA); (ii) managing the logistics of research protocols and data collection under the responsibility of investigators (for a CRT); and (iii) managing a set of research projects with regard to their legal, financial, logistical, administrative, organisational, and human aspects (for a CRPM). The required educational level is practically the same for CRAs and CRTs, either a paramedical or other scientific Bachelor diploma, or a specific CRA/CRT university diploma, for which the training frame is less standardised. In practice, a nurse (Bachelor level) could be hired as a CRA/CRT, and about one-fifth of our sample actually had this qualification. This is a low rate compared to that in countries like Australia, New Zealand, UK, and USA [18–21]. Policy in French hospitals over the last 30 years has been to restrict nurses to care; education in clinical research has been included in the training syllabus of French anaesthetist nurses since 2014 only.

When our participants were asked to define the relationship between the jobs of CRA, CRT, and CRPM, and a list of predefined tasks—which were actually all supposed to fit to the job profile, significant rates of disagreement were observed for certain tasks. Personnel should, of course, be aware of all the tasks related to their jobs, but their differing perception of what they involve could have been due to the very recent publication of the list of tasks [6], and hence a limited awareness of their content. For example, ICU CRTs do not generally have to manage pre-analytical treatment of samples, which is usually done by the hospital laboratory resources. The 30-to-40% rate of inappropriate responses regarding the tasks of CRPMs may have been due to the fact that few of the responders were employed in such a position. Of note, the required educational level to be recruited as a CRPM is higher than for CRAs and CRTs, and most CRPMs are former CRAs/CRTs who have been upgraded after years of experience, as posts for CRPMs have only recently been created. As a result, we lack data on the number of CRPMs in French university public hospitals, but as they are more often located in dedicated offices than embedded in ICUs, this could influence several aspects of their job perception.

Most of the responders had the dual function of CRT and CRA and so had to manage studies sponsored by their own institution (mostly single-centre) and studies with external sponsorship (mostly multicentre). This dual function satisfies the principles of independence (from the sponsor for the CRT, and from the investigators for the CRA), provided one individual does not combine both functions in the same study. Although some institutions are reluctant to allow such multivalence, the current survey showed that it was rather attractive to the personnel, who had a positive perception of it, expressed high satisfaction with the organisation of their personal work, and cited autonomy as the most appreciated positive aspect of the job. The respondents also appreciated the variety in the type of studies they had to work on in terms of setting or sponsoring, and sharing activity with ICUs and other hospital units. This last situation was however specific to general hospitals where one specialty unit is not active enough to involve full-time personnel. Finally, taking part in unit staff meetings was positively perceived and should be encouraged in the ICUs where it is not yet common practice. In this regard, heads of department could act as useful motivators.

Studying job satisfaction, dissatisfaction, and stress can provide suggestions to help retain a specialised workforce such as ICU research personnel. The most cited negative aspects of the job (lack of time, administrative procedures, and paperwork) are probably common to any service job, and therefore hard to act on. However, as already reported in the literature, most attention should be given to how institutions manage careers [19]. Indeed, job satisfaction was average, and the institutional disregard of the needs of CRAs/CRTs and poor recognition of the job within the institution were often cited as sources of stress, and dissatisfaction with career management strongly correlated with job stress.

Some studies performed outside Europe have addressed these issues in research personnel. Two surveys conducted in 2004 and 2009 among 49 research coordinators (RCs) working in Australasian ICUs highlighted the value of autonomy, respect, and intellectual stimulation in the job, while isolation, under-recognition, workload, and under-remuneration were negatively perceived [18, 19]. Of note, we did not address the question of incentive bonuses as they do not really exist in the French public health system. Other teams have also studied the relationships between job satisfaction and personal psychological issues. A nationwide survey conducted in 2005 among 252 RCs in the USA showed that job dissatisfaction was a strong predictor of burnout, while satisfaction was moderately correlated with personal accomplishment [22]. A survey conducted in 2020 among 66 RCs working in Canadian ICUs identified unrealistic workload and weekend/holiday screening as strong stressors, while a positive work environment had the opposite effects [23]. Of note, RCs in the above-mentioned countries have a greater workload than personnel in France, especially on weekends. A study conducted in 2020 among 438 CRAs from 26 major cities across China showed that 82% manifested signs of occupational burnout, of whom half had moderate burnout. The rate of burnout was favoured by mode of working and workload, support provided by the hospital, and the likelihood of receiving a promotion [24]. However, these results could have been different in a period less stressful than that of the COVID pandemic. A study conducted in 2021 among 98 Australasian ICU RCs showed better psychological outcomes, with depression, anxiety, and stress scores within the normal range, and 21 to 27% of the respondents defined as positive to one of these three diagnoses [14]. Conversely, while overall job satisfaction was quite good and close to that in our survey (mean score of 3.5/5), 44% of the respondents exhibited an early symptom of burnout. Unfortunately, we did not assess burnout, but the Maslach Burnout Inventory used in the last two studies above was very sensitive and our observations are consistent with the rest of their results.

Dissatisfaction with training is also correlated with job stress, albeit to a lesser degree, but cannot be neglected because it is closely related to the demand for quality, as stated in the European directive for GCP (chapter 2-1-2) [25]. As our survey showed, educational levels at hiring and training of research personnel from hiring onwards, vary widely. This can be explained by the broad range of required educational levels for new recruits (see paragraph 3 of the Discussion section), a lack of standardisation of the university diplomas specific to CRA/CRT, and the relative newness of the jobs. Although training courses are offered to clinical research personnel in French hospitals, we now need to guarantee that such programs are easily accessible and that they cover all the essential competences, as described in the Joint Task Force for Clinical Trial Competency: scientific concepts and research design; ethics and safety; investigational products; GCP; study/site management; data management; leadership and professionalism; and communications/teamwork [26]. In addition, some ways to improve training need to be addressed in the future, such as having experience in data collection prior to work in monitoring, or being trained specifically to research in an ICU.

The main limitation of the current survey is that it targeted only a sample of the clinical research personnel of French ICUs and not the whole population. The situation in other medical specialties was not addressed either, but such a survey would have been more difficult due to the absence of the extensive network that exists between ICUs.

## Conclusions

To support the employment and career development of ICU research personnel, we hope that our results will be presented to the French Ministry of Health by representatives of academic clinical research, and that they will inspire stakeholders in clinical research in other countries. Among the ways to improve quality and efficiency and to retain this specialised workforce, our survey highlights respect of autonomy, work-sharing between CRAs and CRTs, better career progression, financial reward for demanding tasks, and the opportunity to participate in unit staff meetings.

### Supplementary Information

**Additional file 1**. Details of the survey questionnaire.

**Additional file 2**. Description of the 19 registered participants who did not fully complete the survey.

**Additional file 3**. Details of the tasks currently carried out by the responders (N = 78).

**Additional file 4**. Details of the responders’ suggestions to improve the different aspects of their job (N = 78).

**Additional file 5**. Details of the possible sources of stress identified by the responders (N = 78).

## Data Availability

Not applicable.

## Abbreviations

CRA: Clinical research associate/assistant
CRPM: Clinical research project manager
CRT: Clinical research technician
GCP: Good Clinical Practice
ICH: International Council for Harmonisation
ICM: Intensive care medicine
ICU: Intensive care unit
RC: Research coordinator
