# Supplementary material for: Job description and perception of clinical research personnel working in a network of French intensive care units
Source: Crit Care. 2024 Apr 11;28:119. doi: 10.1186/s13054-024-04900-8 (PMC11010361; doi:10.1186/s13054-024-04900-8)
Supplement: Supplementary file 1 — Additional file 1. Details of the survey questionnaire. [file 13054_2024_4900_MOESM1_ESM.docx]

**Additional file 1. Details of the survey questionnaire.**

**Introduction**

Dear Sir/Madam

You are currently working in an intensive care unit as a member of the clinical research staff.

Here is some information about a survey you are invited to take part in. Read carefully this information note, which will explain the aim of the survey and what it implies for you. Please feel free to ask any questions you may have.

*Background and objective of the study*

The French Ministry of Health’s circular of November 18, 1992, enabled hospitals to set up a management and governance structure for clinical research activities. Within this framework, clinical research in French hospitals has considerably developed over the last 30 years. To facilitate this activity, hospitals have called on multi-disciplinary technical skills acquired in new jobs. To date, however, very little work has been done on the organisation and management of these new activities. Now that the COVID-19 pandemic, during which clinical research played its role alongside care, has ended, we are taking the opportunity to assess the job perception of clinical research staff by those involved in French intensive care units. The aim of this study is to describe the organisation and management of human resources for clinical research in French intensive care units by a nationwide survey of professionals.

*How does the study work?*

If you agree to participate, you will be asked to answer a questionnaire covering the following points:

- your personal and professional profile;
- what you know about the basic skills required;
- the functions you perform;
- the continuing education opportunities available to you;
- your career prospects.

A link to the online questionnaire (to be completed anonymously in 15-20 minutes maximum) will be e-mailed to each identified respondent. If no response is received after 6 weeks, a reminder will be sent by e-mail, followed by telephone contact if necessary.

Data will be collected in real time as soon as the questionnaire is completed, then exported to an Excel file (Microsoft Excel 2010, Microsoft, Redmond, WA, USA) for use by the person in charge of analysis (C. Dualé).

Indicate at the bottom of this information note whether you accept or refuse to take part in this survey. Acceptance will open the survey form, the online completion of which will be proof of your participation.

You may exercise your right to withdraw from this survey at any time without having to justify your decision.

At any time, you can ask the person in charge, (M. Adda), for further information about the study. Finally, you will be informed of the overall results of this research at the end of the study.

Thank you for your attention and participation!

| **Text format or symbols for the data manager** | **Guidelines for reading the survey content** |
| --- | --- |
| typed in black | To include in the questionnaire |
| *typed in black italics* | *List of offered responses (separated by ‘/’)* |
| typed in blue | Title of the section |
| typed in red | Only for information purposes (do not insert) |
| 🡪 | The proposal is conditioned by the response to the previous question [description of the conditional response] |
| 🡨 | End of the conditional part |
| 🗎 | Several possible responses |
| 🗷 | Cell to tick |
| ➊ | Only one possible response (“radio button”) |
| **☯** | Ditto, in binary yes/no format |
| **Ñ** | Numerical value (constrained format) |
| **Ñ%** | Ditto, with a ‘%’ symbol at the end of each line |
| **⮾** | Forced accumulation percentage (pie chart) |
| 🡽 | Storage (sliding or constrained rows) |
| ⯇⯈ | Numerical rating scale |
| **🗀** | Free text |
| **‖‖** | The results for this question are not given in the current report |

Questionnaire completion date: dd/mm/yyyy (will be done automatically)

Job abbreviations and meanings:

- CRA: clinical research assistant (*assistant de recherche clinique*);
- CRPM: clinical research project manager; (*chef(fe) de projet de recherche clinique*)
- CRT: clinical research technician (*technicien de recherche clinique*);
- FTE: full-time equivalent, i.e. legal maximum working time, which is currently 35 hours a week in France, expressed as 1 FTE or 100% FTE.

**Your profile**

You are **☯** *a woman / a man*

Your age group is ➊ *<30 / 30-to-39 / 40-to-49 / 50-to-59 / >60*

Your civil status **☯** *single / domestic partnership/with a partner*

Number of dependent children (*including joint custody*) **Ñ**

Your current working hours (as % of full-time) ➊ *100% / 80% / 50% / other*

**🡪** [other] specify **Ñ** 🡨

Number of teleworking days a week **Ñ**

**Your workplace profile**

Institution where you work (if shared, indicate the main one) ➊ *university hospital* / *general hospital* / *university* *cancer centre* / *other public hospital service* / *other*

**🡪** [other] specify **🗀** 🡨

**‖‖** Department where you work (if shared, indicate the main one) ➊ *medical intensive care* / *multi-purpose intensive care* / *surgical intensive care* / *unspecified “intensive care unit”* / *continuous care* / *other*

**🡪** [other] specify **🗀** 🡨

From this point onwards, the words “in the department” implicitly mean the department where you work (the main department if shared).

**‖‖** Number of beds classified as “intensive care” or “continuous care” in the department (if shared, indicate the principal) **Ñ**

Current number of CRA/CRTs working in the department (in FTE) **Ñ**

Current number of CRPMs working in the department (in FTE) **Ñ**

Current number of active studies in the department, with external sponsorship **Ñ**

Current number of active studies in the department, sponsored by your institution **Ñ**

**Your training**

Did you obtain the *baccalauréat* (or equivalent)? **☯** (NB: equivalent to A level in the UK or High School Diploma in the USA)

When you took up your first job as CRA or CRT, what was your highest educational level? ➊ *1^st^ year Bachelor’s* *degree* / *2^nd^ year Bachelor’s* *degree* / *3^rd^ year Bachelor’s* *degree* / *1^st^ year Master’s* / *2^nd^ year Master’s* / *Doctorate* / *other*

**🡪** [other] specify **🗀** 🡨

What is now your highest level of education? ➊ *1^st^ year Bachelor’s* *degree* / *2^nd^ year Bachelor’s* *degree* / *3^rd^ year Bachelor’s* *degree* / *1^st^ year Master’s* / *2^nd^ year Master’s* / *Doctorate* / *other*

**🡪** [other] specify **🗀** 🡨

When you took up your first job as CRA or CRT, did you have a specific degree in clinical research? **☯**

**🡪** [yes] **‖‖** If yes, which one(s)? 🗎 *FIEC* / *FARC* / *CESAM* / *other*

**🡪** [other] specify **🗀** 🡨

**Your job experience**

Considering your entire career in clinical research (not only your current job), how many years of experience do you have?

- overall **Ñ**
- by function (several functions may overlap at any one time) :

| as a CRA | 🗷 | **🡪**[ticked] **Ñ** |
| --- | --- | --- |
| as a CRT | 🗷 | **🡪**[ticked] **Ñ** |
| as a CRPM | 🗷 | **🡪**[ticked] **Ñ** |
| in another function | 🗷 | **🡪**[ticked] **Ñ** |

In the institution where you work, how many years of experience do you have?

- overall **Ñ**
- by function (several functions may overlap at any one time) :

| as a CRA | 🗷 | **🡪**[ticked] **Ñ** |
| --- | --- | --- |
| as a CRT | 🗷 | **🡪**[ticked] **Ñ** |
| as a CRPM | 🗷 | **🡪**[ticked] **Ñ** |
| in another function | 🗷 | **🡪**[ticked] **Ñ** |

How many years have you worked in clinical research in intensive care? **Ñ**

What function(s) do you currently hold and since when?

| as a CRA | 🗷 | **🡪**[ticked] **Ñ** |
| --- | --- | --- |
| as a CRT | 🗷 | **🡪**[ticked] **Ñ** |
| as a CRPM | 🗷 | **🡪**[ticked] **Ñ** |
| on another function | 🗷 | **🡪**[ticked] **Ñ** |

**How you define the job**

How would you define, for each of these following tasks, the relationship with the **job of CRA**?

For each item the response is ➊ *major* / *minor* / *beyond job scope* / *do not know*

| Designing and building tools or methods |
| --- |
| Checking application of rules, procedures, norms and standards |
| Checking conformity/validity of documents |
| Checking logistical feasibility of the study |
| Checking and monitoring the quality of process(es) |
| Organising events such as meetings, visits, and specialised committees |
| Drafting reports on observations/interventions |
| Monitoring adverse events |

How would you define, for each of these following tasks, the relationship with the **job of CRT**?

For each item the response is ➊ *major* / *minor* / *beyond job scope* / *do not know*

| Checking feasibility of the study’s logistical circuits |
| --- |
| Establishing/updating, and implementing processes, procedures, protocols, and instructions |
| Managing (browsing, collecting, analysing, prioritising, sharing, filing, tracking) data and information |
| Informing/advising the caring staff, patients, families, etc. |
| Organising data checking before monitoring visits |
| Preparing biological sampling, storage and shipping |
| Assessing and presenting the clinical activity of the unit |
| Gathering data or information |
| Completing documents and files (activity or traceability sheets, etc.) |
| Reprography, anonymising results, and transmitting data to the coordinating centre |
| Reporting/tracking adverse events |
| Dispatching study documents (recording, sorting, processing, distribution, archiving) |
| Pre-analytical treatment of samples |

How would you define, for each of these following tasks, the relationship with the **job of CRPM**?

For each item the response is ➊ *major* / *minor* / *beyond job scope* / *do not know*

| Checking application of rules, procedures, norms and standards |
| --- |
| Checking meeting of deadlines (products, files, interventions, etc.) |
| Supervising team(s), staff management and implementation |
| Establishing/updating and implementing processes, procedures, protocols, and instructions |
| Drawing up specifications according to customer’s requirements |
| Planning activities and resources; control and reporting |
| Finding/managing the financial, human and logistical resources |
| Managing grant application files and calls for tender |

**Your job organisation**

In your current job, how are these different functions distributed? (as a percentage of your total activity in clinical research, whatever the total FTE; the sum must be 100%) **⮾**

- *CRA* **Ñ%**
- *CRT* **Ñ%**
- *CRPM* **Ñ%**
- *other* **Ñ%**

If your job is multivalent, what do you think of it? *Not applicable (monovalent)* 🗷

**🡪**[not ticked] ➊ *very satisfactory* / *satisfactory* / *neutral opinion or no opinion* / *unsatisfactory* / *not at all satisfactory* 🡨

In general, how many studies sponsored by your institution do you have to manage at the same time? ➊ *none* / *1-to-2* / *3-to-4* / *more than 4*

In general, how many studies sponsored by an external institution do you have to manage at the same time? ➊ *none* / *1-to-5* / *5-to-10* / *more than 10*

Of all the studies you are in charge of, how many are multicentre? **Ñ**

Are you called upon at night or at weekends in the course of your work? **☯**

Is this provision included in your contract? **☯**

In your current job, how are these different tasks distributed? (as a percentage of your total clinical research activity, whatever the total FTE; the sum must be 100%) **⮾**

- *presence in the department (managing inclusions, patient follow-up, etc.)* **Ñ%**
- *in an office (data entry, file management, drafting protocols, articles, procedures, etc.)* **Ñ%.**
- *other* **Ñ%**

For studies sponsored by an external institution, how do you screen eligible patients? 🗎

- *by taking part in the unit staff meetings or in daily visits to patients?*
- *by a regular (ideally daily) meeting with a dedicated medical doctor?*
- *by consulting the patients' medical records on your own?*
- *other (please specify)* **🡪🗀**🡨

If you need quiet time in an office to work, what tasks do you perform there? 🗎

- *Data entry, responses to queries, reporting SAEs*
- *Patient follow-up*
- *Drafting protocols*
- *Drafting procedures*
- *Writing newsletters*
- *Writing articles or reports on results (communications, posters, etc.)*
- *Other (please specify)* **🡪🗀**🡨

How often do you take part in unit staff meetings? ➊ *never* / *weekly* / *bimonthly* / *monthly* / *other*

**🡪** [other] specify **🗀** 🡨

Do you find it useful to take part in unit staff meetings? ☯

**🡪** [yes] If yes, can you rank these aspects from most to least useful? 🡽

- *Facilitating identification or selection of patients for inclusion*
- *Learning/expanding medical vocabulary*
- *Improved knowledge of diseases and care*
- *Better integration in the team* 🡨

Among the positive aspects of your job listed below, can you rank them, from the one you appreciate the most to the one you appreciate the least? 🡽

- *Autonomy*
- *Relational aspects*
- *Working in a team*
- *Organisation and logistical management*
- *Scientific interest*
- *Personal development*

Of the negative aspects of your job listed below, can you rank them, from the one you dislike the most to the one you dislike the least? 🡽

- *Lack of time*
- *Routine*
- *Isolation*
- *Constraints*
- *Administrative procedures and paperwork*

**Your career**

**‖‖** When you were hired as a CRA/CRT in your current institution, what was your administrative status? ➊ *senior hospital technician* / *hospital engineer* / *psychologist* / *nurse* / *other*

**🡪** [other] specify **🗀** 🡨

**‖‖** When you were hired as a CRA/CRT in your current institution, what was the type of contract? ➊ *temporary with definite duration* / *temporary with indefinite duration* / *tenure*

What type of contract are you currently on? ➊ *temporary with definite duration* / *temporary with indefinite duration* / *tenure*

**🡪** [tenure] How many months did the contract last before you obtained tenure? **Ñ** 🡨

**‖‖** Who is currently your direct supervisor? ➊ *the institution’s director for clinical research* / *the head of department* / *a CRPM* / *other*

**🡪** [other] specify **🗀** 🡨

Do you currently work in several different care units? ☯

**🡪**[yes]

In how many departments? **Ñ**

In how many intensive care units? **Ñ**

For what proportion of time in intensive care? **Ñ%** 🡨

Do you have appraisal meetings with your supervisor? ☯

**🡪** [yes] If yes, how often? ➊ *half-yearly* / *yearly* / *biennial* / *other*

**🡪** [other] specify **🗀** 🡨

Does your institution offer you prospects for career development in your profession? ☯

**🡪** [yes] If yes, which ones? **🗀** 🡨

**How you feel about your work**

Which of the following situations are sources of stress for you personally in your job? 🗎

Functional aspects 🗎

- Personal workload
- Working conditions (e.g. working at night or on weekends)
- Organisational changes in the department
- Organisation of personal trips
- Financial aspects of personal trips (e.g. advanced expenses payment)
- Management and organisational issues
- Understaffing (e.g. because of absences)
- Opaque division of tasks
- Institutional neglect of the needs of CRAs/CRTs
- Institutional outdated or opaque functioning
- Permanent pressure on the units
- Overflow of functions
- Involvement in the search for funding
- Involvement in staff management

Relational aspects 🗎

- Conflicts between research colleagues
- Conflicts with medical doctors
- Conflicts with carers
- Involvement in obtaining patient consent
- Personal difficulties with human contact
- Unequal workload between colleagues
- Isolation from the other units

Social aspects 🗎

- Poor recognition of the job within the institution
- Poor visibility of the profession outside the institution

How satisfied would you say you are with the way your training has gone since you joined your institution?⯇⯈ (*10 for “maximum satisfaction”*)

Which of the following avenues for improvement do you consider interesting? 🗎

- Diploma or institutional training in methodology
- Diploma or institutional training in biostatistics
- Diploma or institutional training in English
- Deepening medical knowledge by attending unit staff meetings
- Information about innovations by meeting medical representatives
- Meetings/forums with professionals from other departments or institutions
- Training courses for doctors and CRAs
- Secondment of clinical research staff to other departments during slack periods
- Other

**🡪** [‘other’ ticked] specify **🗀** 🡨

If your job is multivalent (CRA+CRT, CRA+CRPM, etc.), how would you rate your sense of personal job efficiency?

- *Not applicable (monovalent)* 🗷
- **🡪** [not ticked]⯇⯈ *(10 for “maximum efficiency”)* 🡨

How would you rate the complexity of your institution’s organisation in managing the studies it sponsors?⯇⯈ *(10 for “maximum complexity”)*

Which of the following avenues for improvement do you consider interesting? 🗎

- Define a dedicated team for each medical specialty
- Reduce the number of contacts within the institution’s research department
- Other

**🡪** [‘other’ ticked] specify **🗀** 🡨

How would you rate your satisfaction with the organisation of your work? ⯇⯈ *(10 for “maximum satisfaction”)*

Which of the following areas for improvement do you consider interesting? 🗎

- Business software covering all these aspects
- Other

**🡪** [‘other’ ticked] specify **🗀** 🡨

How would you rate your level of satisfaction with the way your institution manages your career? ⯇⯈ *(10 for “maximum satisfaction”)*

How would you rate your level of satisfaction with the organisation of clinical research within your institution?⯇⯈ *(10 for “maximum satisfaction”)*

Which of the following areas for improvement do you consider interesting? 🗎

- Separating the functions (CRA, CRT, CRPM)
- Providing suitable working conditions (equipment, office…)
- Retaining each CRA/CRT in one given care unit
- Retaining each CRA/CRT in one given speciality
- Financial reward for demanding tasks (bonuses, etc.)
- Other

**🡪** [‘other’ ticked] specify **🗀** 🡨

How would you rate your level of stress in your job?⯇⯈ *(10 for “maximum stress”)*

Do you have any suggestions for reducing or preventing this stress? **🗀**
