# Supplementary material for: Job description and perception of clinical research personnel working in a network of French intensive care units
Source: Crit Care. 2024 Apr 11;28:119. doi: 10.1186/s13054-024-04900-8 (PMC11010361; doi:10.1186/s13054-024-04900-8)
Supplement: Supplementary file 2 — Additional file 2. Description of the 19 registered participants who did not fully complete the survey. [file 13054_2024_4900_MOESM2_ESM.docx]

**Additional file 2. Description of the 19 registered participants who did not fully complete the survey.**

| **Demography** | |
| --- | --- |
| Female | 18 (94.7) |
| Age group | |
| *<30* | 0 (0.0) |
| *30-to-39* | 6 (31.6) |
| *40-to-49* | 9 (47.4) |
| *50-to-59* | 3 (15.8) |
| *>60* | 1 (5.3) |
| In a partnership | 13 (68.4) |
| Number of dependent children (including if joint custody) | |
| *0* | 8 (42.1) |
| *1* | 2 (10.5) |
| *2* | 7 (36.8) |
| *3 or more* | 2 (10.5) |
| **Educational level** | |
| French *baccalauréat* (or equivalent) ^a^ | 16 (94.1) |
| Current highest level of education ^b^ | |
| *no degree* | 1 (5.9) |
| *Bachelor degree ^c^* | 11 (64.7) |
| *Master degree* | 3 (17.6) |
| *PhD* | 2 (11.8) |
| Specific CRA/CRT diploma when taking up the first job in the hospital | 8 (47.1) |
| Number of years’ experience in clinical research | |
| In clinical research, all positions combined | 9 (4 – 12) |
| In the current workplace, all positions combined | 10 (4 – 20) |
| Clinical research in ICU | 4 (4 – 10) |
| **Job description** | |
| Type of hospital | |
| *General* | 8 (42.1) |
| *University* | 11 (57.9) |
| Size of the ICU as workplace (number of beds) | 24 (17 – 27) |
| Number of CRAs/CRTs currently working in the unit (in FTE) | 1 (1 – 3.25) ^d^ |
| Number of ongoing studies in the unit, with external sponsorship | 14 (12 – 20) |
| Number of ongoing studies in the unit, sponsored by own institution | 1 (0.25 – 2) |
| Amount of weekly work (in % of a FTE) | |
| *<20%* | 1 (5.3) |
| *80-to-90%* | 12 (10.5) |
| *100%* | 16 (80.2) |
| Number of weekly teleworking days | |
| *0* | 14 (73.7) |
| *1* | 4 (21.1) |
| *≥2* | 1 (5.3) |

General description of the 19 registered participants who did not fully complete the survey (see Results section for details). Numerical data are expressed as median (1^st^ quartile – 3^rd^ quartile). Nominal data are expressed as headcount (%). Abbreviations: CRA: clinical research assistant/associate (*attaché de recherche clinique*), i.e. working on behalf of the sponsor; CRT: clinical research technician (*technicien de recherche clinique*), i.e. working on behalf of the centre with the investigating team; FTE: full-time equivalent, i.e. the legal maximum working time, which is currently 35 hours a week in France; 1 FTE or 100% FTE corresponds to this duration; ICU: intensive care unit. Notes: a, equivalent to ‘A level’ in the UK or ‘High School Diploma’ in the USA; b, in the French university system, the three levels of graduation (college) are *licence* (Bachelor, 3 years), *master* (2 years) and *doctorat* (i.e., PhD); c, including 3 Bachelors in Nursing; d, maximum = 7.
