# Supplementary material for: Job description and perception of clinical research personnel working in a network of French intensive care units
Source: Crit Care. 2024 Apr 11;28:119. doi: 10.1186/s13054-024-04900-8 (PMC11010361; doi:10.1186/s13054-024-04900-8)
Supplement: Supplementary file 3 — Additional file 3. Details of the tasks currently carried out by the responders (N = 78). [file 13054_2024_4900_MOESM3_ESM.docx]

# **Additional file 3. Details of the tasks currently carried out by the responders (N = 78).**

| Method for screening eligible patients for studies sponsored by an external institution ^a^ | |
| --- | --- |
| *personally consulting the patients’ medical records* | 49 (62.8) |
| *taking part in the unit staff meetings or in daily visits to patients* | 37 (47.4) |
| *regular (ideally daily) meeting with a dedicated medical doctor* | 28 (35.9) |
| *Another method* | 6 (7.7) |
| Frequency of attendance at the unit staff meetings | |
| *daily or almost daily* | 9 (12.3) |
| *weekly* | 26 (35.6) |
| *bimonthly* | 2 (2.7) |
| *monthly* | 3 (4.1) |
| *never or rarely* | 33 (45.2) |
| Tasks performed quietly in an office (if applicable) ^a^ | |
| Data entry, responses to queries, reporting SAEs | 66 (84.6) |
| Patient follow-up | 55 (70.5) |
| Drafting procedures | 49 (62.8) |
| Writing newsletters | 24 (30.8) |
| Drafting protocols | 18 (23.1) |
| Writing articles or reports on results (communications, posters, etc.) | 15 (19.2) |
| Other tasks | 14 (17.9) |

Nominal data are expressed as headcount (%). Abbreviations: SAE, serious adverse event. Notes: a, several possible responses (in this case, the items are ranked by decreasing rate of response).
