# Supplementary material for: Job description and perception of clinical research personnel working in a network of French intensive care units
Source: Crit Care. 2024 Apr 11;28:119. doi: 10.1186/s13054-024-04900-8 (PMC11010361; doi:10.1186/s13054-024-04900-8)
Supplement: Supplementary file 4 — Additional file 4. Details of the responders’ suggestions to improve the different aspects of their job (N = 78). [file 13054_2024_4900_MOESM4_ESM.docx]

# **Additional file 4. Details of the responders’ suggestions to improve the different aspects of their job (N = 78).**

| **Regarding professional training ^a^** | |
| --- | --- |
| Diploma or institutional training in English | 38 (48.7) |
| Meetings/forums with professionals from other departments or institutions | 28 (35.9) |
| Training courses for doctors and CRAs | 25 (32.1) |
| Diploma or institutional training in methodology | 24 (30.8) |
| Diploma or institutional training in biostatistics | 18 (23.1) |
| Deepening medical knowledge by attending unit staff meetings | 16 (20.5) |
| Secondment of clinical research staff to other departments during slack periods | 10 (12.8) |
| Information about innovations by meeting medical representatives | 7 (9.0) |
| **Regarding** **clinical research structures ^a^** | |
| Reduce the number of contacts within the institution’s research department | 36 (46.2) |
| Define a dedicated team for each medical speciality | 33 (42.3) |
| **Regarding** **personal work organisation** | |
| Business software covering all these aspects | 39 (50.0) |
| **Regarding career management ^a^** | |
| Financial reward for demanding tasks (bonuses, etc.) | 59 (75.6) |
| Providing suitable working conditions (equipment, office…) | 35 (44.9) |
| Retaining each CRA/CRT in one given care unit | 29 (37.2) |
| Retaining each CRA/CRT in one given speciality | 24 (30.8) |
| Separating the functions (CRA, CRT, project manager) | 14 (17.9) |

Nominal data are expressed as headcount (%). Notes: a, several possible responses (in this case, the items are ranked by decreasing rate of response). Abbreviations: CRA: clinical research assistant/associate (*attaché de recherche clinique*), i.e. working on behalf of the sponsor; CRT: clinical research technician (*technicien de recherche clinique*), i.e. working on behalf of the centre with the investigating team.
