# Supplementary material for: Job description and perception of clinical research personnel working in a network of French intensive care units
Source: Crit Care. 2024 Apr 11;28:119. doi: 10.1186/s13054-024-04900-8 (PMC11010361; doi:10.1186/s13054-024-04900-8)
Supplement: Supplementary file 5 — Additional file 5. Details of the possible sources of stress identified by the responders (N = 78). [file 13054_2024_4900_MOESM5_ESM.docx]

# **Additional file 5. Details of the possible sources of stress identified by the responders (N = 78).**

| **Functional aspects** | |
| --- | --- |
| Institutional neglect of the needs for CRAs/CRTs | 39 (50.0) |
| Personal workload | 31 (39.7) |
| Understaffing (e.g. because of absences) | 28 (35.9) |
| Institutional archaic or opaque functioning | 25 (32.1) |
| Overflow of functions | 23 (29.5) |
| Management and organisational issues | 21 (26.9) |
| Permanent pressure on the units | 14 (17.9) |
| Working conditions (e.g. work at night or on weekend) | 10 (12.8) |
| Organisation of personal travels | 10 (12.8) |
| Financial aspects of personal travels (e.g. advanced expenses payment) | 10 (12.8) |
| Opaque division of tasks | 10 (12.8) |
| Organisational changes in the department | 9 (11.5) |
| Involvement in the search for funding | 8 (10.3) |
| Involvement in staff management | 6 (7.7) |
| **Relational aspects** | |
| Unequal workload between colleagues | 22 (28.2) |
| Isolation from the other units | 19 (24.4) |
| Conflicts between research colleagues | 14 (17.9) |
| Involvement in obtaining the patients’ consent | 14 (17.9) |
| Conflicts with carers | 10 (12.8) |
| Conflicts with medical doctors | 4 (5.1) |
| **Social aspects** | |
| Poor recognition of the job within the institution | 47 (60.3) |
| Poor visibility of the profession outside the institution | 31 (39.7) |

Nominal data are expressed as headcount (%). Within each domain, there were several possible responses; the items are ranked by decreasing rate of response. Abbreviations: CRA: clinical research assistant/associate (*attaché de recherche clinique*), i.e. working on behalf of the sponsor; CRT: clinical research technician (*technicien de recherche clinique*), i.e. working on behalf of the centre with the investigating team.
